# Elevated Anthropometric and Metabolic Indicators among Young Adult Offspring of Mothers with Pregestational Diabetes: Early Results from the Transgenerational Effect on Adult Morbidity Study (the TEAM Study)

**DOI:** 10.1155/2021/6590431

**Published:** 2021-11-01

**Authors:** Katherine Bowers, Shelley Ehrlich, Lawrence M. Dolan, Resmi Gupta, Mekibib Altaye, Nicholas J. Ollberding, Rhonda Szczesniak, Patrick Catalano, Emily Smith, Jane C. Khoury

**Affiliations:** ^1^Division of Biostatistics and Epidemiology, Cincinnati Children's Hospital Medical Center, Cincinnati, Ohio, USA; ^2^University of Cincinnati College of Medicine, Department of Pediatrics, Cincinnati, Ohio, USA; ^3^Division of Endocrinology, Cincinnati Children's Hospital Medical Center, Cincinnati, Ohio, USA; ^4^Biostatistics and Epidemiology/Research Design Component, Division of Clinical and Translational Sciences, Department of Internal Medicine, University of Texas McGovern Medical School, Houston, Texas, USA; ^5^Tufts University School of Medicine, Friedman School of Nutrition, Science and Policy, Boston, Massachusetts, USA

## Abstract

Exposure to maternal diabetes *in utero* increases the risk in the offspring for a range of metabolic disturbances. However, the timing and variability of *in utero* hyperglycemic exposure necessary to cause impairment have not been elucidated. The TEAM Study was initiated to evaluate young adult offspring of mothers with pregestational diabetes mellitus. This paper outlines the unique enrollment challenges of the TEAM Study and preliminary analysis of the association between exposure to diabetes in pregnancy and adverse metabolic outcomes. The TEAM Study enrolls offspring of women who participated in a Diabetes in Pregnancy (DiP) Program Project Grant between 1978 and 1995. The DiP Study collected medical and obstetric data across pregnancy. The first 96 eligible offspring of women with pregestational diabetes were age-, sex-, and race-matched to adults from the National Health and Nutrition Examination Survey (NHANES) 2015-2016 with an OGTT. Descriptive and regression analyses were employed to compare TEAM participants to NHANES participants. Among a subset of TEAM participants, we compared the metabolic outcomes across maternal glucose profiles using a longitudinal data clustering technique that characterizes level and variability, in maternal glucose across pregnancy. By comparing categories of BMI, TEAM Study participants had over 2.0 times the odds of being obese compared to matched NHANES participants (for class III obesity, OR = 2.81; 95% confidence interval (CI): 1.15, 6.87). Increasing levels of two-hour glucose were also associated with *in utero* exposure to pregestational diabetes in matched analyses. Exposure to pregestational diabetes *in utero* may be associated with an increased risk of metabolic impairment in the offspring with clinical implications.

## 1. Introduction

Diabetes mellitus has reached epidemic proportions in the United States and around the world. In some counties in the United States, over 25% of the population has diabetes [1] and 35% of adults 20 years and older have prediabetes [2]. Furthermore, the prevalence of diabetes has increased among women of child-bearing age [3]. Even in regions with the lowest prevalence, nearly one-tenth of the population is affected [1]. Not only is diabetes itself the seventh leading cause of death but also the consequences of diabetes can be transferred to the next generation.

Exposure to maternal diabetes in utero increases the risk in the offspring for metabolic disturbances, including obesity [4–7], insulin resistance [8–10], type 2 diabetes mellitus [6, 11, 12], and cardiovascular (CV) dysfunction [13, 14]. In addition to metabolic consequences, offspring of mothers with diabetes may be at risk for cognitive and behavioral impairments [15, 16].

While these associations are clear, the timing of hyperglycemic exposure across pregnancy, as well as the level and variability of exposure necessary to cause impairment, has not been elucidated. In addition, it is unknown whether detection of more subtle health consequences, early in the natural history, may provide opportunities for secondary prevention.

In an effort to fill the gap regarding the level and timing of diabetic hyperglycemia in utero, the Transgenerational Effect on Adult Morbidity (TEAM) Study was initiated to evaluate young adult offspring of mothers with pregestational diabetes mellitus, type 1 diabetes or type 2 diabetes, to determine the association between the timing and variability of glucose exposure in pregnancy and risk of obesity, diabetes, and renal and cardiovascular compromise in adult offspring. Building on a Program Project Grant, herein referred to as the Diabetes in Pregnancy (DiP) Study, conducted at the University of Cincinnati Medical Center and Cincinnati Children's Hospital Medical Center between 1978 and 1995, the TEAM Study is enrolling up to 250 young adults from the 454 offspring of women with pregestational diabetes who participated in the DiP Study. The objective of the TEAM Study is to evaluate the association between hyperglycemia in pregnancy and biomarkers, intermediates, and clinical outcomes related to metabolic, cardiac, nephrotic, and both cognitive and behavioral outcomes (Table 1).

The DiP Study examined the effect of the level of maternal diabetic control on major congenital malformations in offspring. This landmark study, along with others [17, 18], demonstrated the benefit of strict glucose control throughout pregnancy resulting in a decreased incidence of congenital malformations and of perinatal mortality from 17% and 16% [19], respectively, to rates which approach those for pregnancies not complicated by diabetes (around 3% and less than 10 per 1,000, respectively) [20–22]. However, exposure to hyperglycemia may result in more subtle and long-term effects to offspring, motivating the initiation of the TEAM Study. The DiP Study collected comprehensive longitudinal clinical, obstetric, and perinatal data throughout pregnancy and delivery (described below), which will be leveraged for the TEAM Study.

In order to enroll offspring of mothers who participated in the DiP Study, it is necessary to identify, locate, and acquire contact information and then contact and enroll individuals with whom the study has had no prior contact and whose mothers have not been contacted in up to 43 years. This is a formidable task but once completed will culminate in an unparalleled research opportunity. Nearly two years into recruiting, over 100 offspring have been enrolled and over 400 have been identified.

This paper describes the successful methods addressing each of the challenges of identifying and enrolling the participants. In addition, we describe the comprehensive TEAM Study procedures, provide a description of the cohort to date, and present preliminary analyses. Preliminary analyses presented here compare anthropometric and metabolic outcomes of TEAM participants (exposed to pregestational diabetes *in* utero) to an age-, sex-, and race-matched cohort from the National Health and Nutrition Examination Survey (NHANES). In addition, among a subset of participants, we examine associations in the mean level and variability of glucose across pregnancy, characterized by maternal glucose profiles.

## 2. Methods

### 2.1. Diabetes in the Pregnancy Program Project Grant (DiP)

The DiP Study was a clinical trial conducted at the University of Cincinnati between 1978 and 1995, which enrolled women preconceptionally or during pregnancy prior to 10-week gestation for randomization and later in gestation for observation. Participants were diagnosed with diabetes prepregnancy including women with both type 1 and type 2 diabetes. The purpose of the study was to determine if more strict glucose targets combined with more frequent clinic visits early in pregnancy would have an impact on pregnancy outcomes. Women participating in the clinical trial were randomized to receive either strict glycemic control or customary glycemic control [22]. Treatment groups were defined by fasting and 90-minute targeted levels of glucose control. Fasting and 90-minute postprandial blood glucose targets for strict glycemic control were <100 mg/dL and<120 mg/dL, respectively, and those for customary glycemic control were <120 mg/dL and <140 mg/dL, respectively [23]. Blood glucose was monitored at clinic visits and daily by participants (after 1981). At the clinic visit, both pre- and 90-minute postprandial blood glucose concentrations were measured. At home (after 1981), reflectance blood glucose meters (Ames Dextrometer; Miles Inc., Diagnostics Division, Elkhart, IN) were employed for women to self-monitor glucose levels four to six times daily.

Complete medical and obstetric histories were obtained from each participant. Ongoing medical data related to diabetes and pregnancy were obtained at regular clinic visits, which were required for study participation. During the first trimester, clinic visits occurred every week for the strict glycemic control group and every 2 weeks for the standard control group. For the rest of pregnancy, all participants had weekly clinic visits. Care at each visit was provided by a team of specialists including a dietician, a diabetes nurse educator, a maternal-fetal specialist, and an endocrinologist with additional care available by an ophthalmologist, neonatologist, and geneticist. Women were provided intensive diabetes education, and their glucose control and insulin requirements were monitored throughout gestation to optimize management of their diabetes. Infants, whether liveborn or stillborn, were examined by both a neonatologist and geneticist/dysmorphologist immediately after birth.

### 2.2. The TEAM Study

The TEAM Study is aimed at applying innovative statistical approaches to associate the timing, level, and variability of *in utero* glucose exposure to morbidity in the adult offspring of women with pregestational diabetes mellitus. The specific aims of the TEAM Study are as follows:

1. To demonstrate the transgenerational effect of the hyperglycemic intrauterine environment on metabolic health of adult offspring of women with pregestational diabetes. Specific gestational periods of hyperglycemia predictive of specific metabolic morbidities in the adult offspring will be identified
2. To demonstrate the transgenerational effect of the hyperglycemic intrauterine environment on cardiac and peripheral vascular structure and function in adult offspring of women with pregestational diabetes. We will determine if cardiovascular compromise in the adult offspring may be predicted by the gestational glycemic profile
3. To determine the effect of the hyperglycemic intrauterine environment on cognition in young adult offspring of women with pregestational diabetes
4. To determine the effect of exposure to a hyperglycemic intrauterine environment on behavioral outcomes in young adult offspring of women with pregestational diabetes

### 2.3. The TEAM Study Procedures

The TEAM Study includes one clinical study visit that takes place at the Cincinnati Children's Hospital Medical Center (CCHMC) William K. Shubert Clinical Research Center. Participants are asked to fast for 9 hours prior to the visit and provide first morning urine using a collection kit that was provided in advance. Upon arrival, participants provide a second urine sample to assess renal function and to test for pregnancy in female participants. If they are pregnant, they are required to reschedule at least 3 months following the pregnancy outcome. Participants undergo anthropometric measures and cardiovascular tests of structure and function including left ventricular mass (LVM), pulse wave velocity (PWV), augmentation index, and carotid intima-media thickness (cIMT) to enable detection of subclinical abnormalities of cardiac and peripheral vascular structure and function; a fasting blood sample and a frequently sampled oral glucose tolerance test (OGTT) (glucose measured by an enzymatic assay) and c-peptide were interpreted using the minimal model developed by Gower et al. [24, 25] to assess beta cell function; dual-energy X-ray absorptiometry (DXA) and sagittal abdominal diameter (SAD) to assess visceral and total body fat, measurement of hip, and iliac and midpoint waist; measures of nutrition, physical activity, and a sleep survey, as well as neurocognitive and behavioral testing (the fasting blood sample also provides measures of metabolic, cardiac, and renal indices).

#### 2.3.1. Potential Participant Identification and Contact Process

The TEAM Study sampling base was limited to the 454 offspring of DiP pregnancies. We aim to enroll 250 of these offspring for the TEAM Study. The youngest enrolled participant will be 22 years old and the oldest up to 43 years old at the time of study participation. To ensure an unbiased order of recruitment, the list of the 435 offspring (all eligible offspring except 19 who participated in a pilot study in 2008/2009) was randomized using simple randomization to determine the order of contact. The identity of most offspring was unknown as infant names were not recorded in the DiP Study database. We therefore employed two approaches to identify and contact the offspring: (1) contacting their mothers and (2) a comprehensive Internet search for names and contacts using methods described below.

To contact mothers, starting with the last known contact, a letter was sent describing the TEAM Study and asking for contact information for their offspring. However, for some mothers, only mothers' name and both her date of birth and that of the offspring were available, so a comprehensive Internet search was conducted to find her current contact information employing many of the methods listed below. In addition, if mothers were found to be deceased, a search was conducted to locate an obituary which could potentially include the offspring names.

To determine the identity of offspring directly, the following procedures were employed in order:

- The CCHMC electronic medical record (EPIC) was searched using the infants' date of birth and sex. Each date of birth/sex search resulted in approximately 30 records to be reviewed for matches with the mothers' name
- Accurint LexisNexis was searched for the mother using her name and date of birth. Though LexisNexis does not track relatives, this provided information about the mothers' current city
- Using the mother's current name and city, a search of https://fastpeoplesearch.com/ was employed which results in a current age (additional confirmation of correct individuals) and family members. These family members were reviewed to identify the offspring with an age or date of birth and sex matching those of the offspring in question
- The offspring name was searched in LexisNexis to confirm age and date of birth or determine if they are incarcerated or deceased
- If still unable to identify and locate participants, additional websites were searched including http://familytreenow.com/ (which lists all possible relatives and year they were born) and Google searches

Once potential participants were identified, contact was initiated to introduce the study and confirm interest in participation. First, letters were sent with a contact information sheet, business reply, and refusal opt out. The study team then waited six weeks until additional communications were attempted. After six weeks, participants were called using phone numbers identified through online searches. The frequency of calls was every 8-15 days. After four voice mails were left with no return call, the frequency was reduced to once per month or ceased for a period of time to focus on the next batch. If only an email was identified online, an email was sent every two weeks for about six weeks and then likewise ceased for a period of time to focus on the next batch. If no phone number or email address was available, then a follow-up contact attempt from the letter was not possible. Post cards were also sent to 88 potential participants who never made verbal contact or for whom the study team had sent information and had been attempting contact for more than three months.

To date these methods have been successful for identifying our first 107 participants who completed a study visit prior to the March 2020 COVID-19 shutdown. However, to contact the next set of potential participants, additional methods will be employed, including additional contact via phone calls and emails to the original DiP participants, the mothers. It was a priori determined to concentrate on contacting offspring of mothers with type 1 diabetes first and then mothers with type 2 diabetes, and this was included in the randomization scheme. Thus, the first 96 (after excluding 11 participants who were taking insulin or other oral or injectable medications for diabetes in order to replicate exclusion criteria used for the NHANES OGTT) included in the analyses are offspring of mothers with type 1 diabetes only. All participants provided written informed consent.

### 2.4. Preliminary Analysis

#### 2.4.1. NHANES

Analyses were conducted using the 2015-2016 NHANES cohort, the most current data available at the time of analysis. NHANES participants were eligible for inclusion in the present analyses if they provided fasting and 2-hour samples for the oral glucose tolerance test (OGTT). In addition, NHANES participants included for matching were restricted with the same age range as TEAM participants (24-43 years). In total, 719 individuals were included from the NHANES cohort. For NHANES, the fasting blood tests were performed on all participants who were over 12 years of age following a nine-hour fast. For the OGTT, after initial venipuncture, participants consumed a 75 g dose of glucose (Trutol™). After two hours, a second venipuncture was performed. Participants were excluded from the OGTT if they had hemophilia, were on chemotherapy, had fasted less than nine hours, and were taking insulin or other oral or injectable medications for diabetes, if they had self-reported weight loss or bariatric surgery, and if they refused phlebotomy, were pregnant, or were unable to consume the Trutol™ in the allotted time (5 minutes). Glucose was measured employing an enzymatic method using the Roche C311 (https://wwwn.cdc.gov/Nchs/Nhanes/2015-2016/OGTT_I.htm), and HbA1c was measured using the Tosoh Automated Glycohemoglobin Analyzer HLC-723G8 for quantitative measurement of the percent HbA1c in whole blood (https://wwwn.cdc.gov/nchs/data/nhanes/2015-2016/labmethods/GHB_I_MET.pdf).

#### 2.4.2. Matching of TEAM Study Participants with NHANES Participants

TEAM Study participants were age- (within 1 year), sex-, and race-matched up to 1 : 3 to NHANES participants using the gmatch macro for SAS, which employs a greedy matching algorithm, also known as the local optimal method [26]. Using the greedy method, after randomly sorting each group, a match is selected once a participant is identified meeting the matching criteria and is not broken, even if more optimal matches could be found across the sample. The “distance” between TEAM Study and comparison participants (*D*_*ij*_) was determined by identifying the NHANES participant (*j*) closest to the TEAM participant (*i*) based on the weighted sum of the absolute difference between the matching factors. This process is repeated until no more matches can be found up to the preselected case to the comparison ratio within the program.

#### 2.4.3. Outcomes

The primary outcomes included body mass index (BMI, kg/m^2^), obesity class (normal: BMI < 25; overweight: 25 ≤ BMI < 30; class I:30 ≤ BMI < 35; class II: 35 ≤ BMI < 40; and class III: BMI ≥ 40), iliac waist circumference (mean centimeters of three measurements), systolic blood pressure (SBP, mean mmHg of three measurements), diastolic blood pressure (DBP, mean mmHg of three measurements), fasting glucose (<100 mg/dL, 100-<126 mg/dL, and ≥126 mg/dL), and 2-hour glucose (<140 mg/dL, 140-<200 mg/dL, and ≥200 mg/dL) and HbA1C (<5.7%/39 mmol/mol, 5.7-6.4%/39 mmol/mol–46 mmol/mol, and ≥6.5%/48 mmol/mol).

#### 2.4.4. Statistical Analyses

Data were summarized using *n* (%) for categorical variables and means (standard deviations) and medians (25^th^-75^th^ percentile) for continuous variables. Differences were evaluated using linear regression, accounting for the matched sets by employing a random effect of an identity variable for each matched cluster and for continuous variables and a Friedman test for categorical variables. Logistic regression employing GEE to the matched sets generated odds ratios and 95% confidence intervals describing the odds of metabolic impairment in TEAM participants versus the comparison (NHANES) participants.

Among a subset of TEAM participants whose mothers provided up to six daily glucose measures, glucose profiles representing longitudinal patterns of control across pregnancy were evaluated [27]. Profiles of temporal glucose were estimated utilizing cubic B-splines. Sparse functional principal component analysis (fPCA) for longitudinal data was used to obtain univariate scores based on the first fPC. Each mother's score was used to assign her profile into exactly one of three groups. Based on these scores, those below the first quartile of scores were classified as group 1 and represented high mean and variability; those between the first and third quartile of scores were classified as group 2 and represented moderate mean levels with moderate variability; scores exceeding the third quartile were considered to be in group 3 and represented low mean and variability across pregnancy [27]. ANOVA and chi-square tests determined whether differences in continuous and categorical variables, respectively, varied across glucose profile groups.

## 3. Results

**Figure 1:**
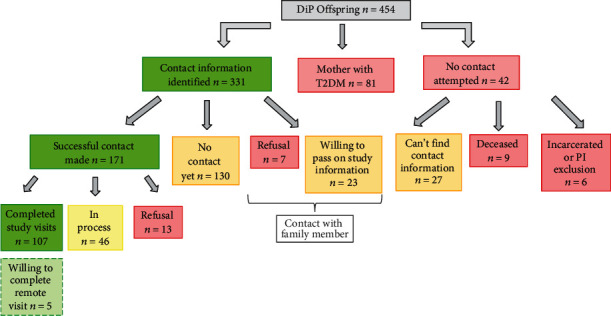
Eligible participant identification, contact attempts, and enrollment in the TEAM Study.

The first mailing was initiated on February 15, 2018, and the 107^th^ participant was enrolled on February 11, 2020. Contact information was identified for 331 of the 454 offspring through either contact with the original DiP participant (mother) or directly searching for the participants. Of the 331, there has been successful contact with 171 participants (no successful contact yet with 130 individuals). For the remaining 30 individuals, we have successfully contacted a family member. For seven of the 30, there was a refusal by proxy (unwilling to share information) while 23 were willing to either pass along study information or provide the participant's information. In total, 107 study visits have been completed, 46 are in-process (scheduled or will be recontacted for scheduling), and 13 individuals refused study participation. There have been 5 participants willing to schedule a remote visit, which is planned to take place in the coming year. There are 123 offspring with whom no contact has been attempted, 81 of whom are offspring of women with type 2 DM. For the remaining 42 offspring of mothers with type 1 DM, we have been unable to find their name or contact information online for 27; however, of the other 15, nine are deceased and six are either incarcerated or were excluded at the PI discretion, but no other exclusion criteria were applied (Figure 1).

**Figure 2:**
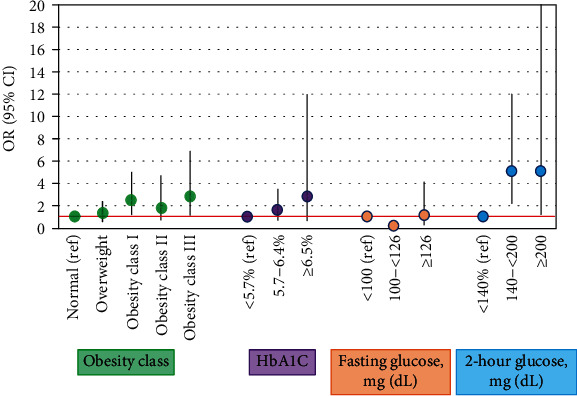
Odds ratios (95% confidence intervals) for adverse anthropometric and metabolic outcomes comparing offspring of mothers with pregestational diabetes (the TEAM Study) to age-, sex-, and race-matched NHANES 2015-2016 participants.

The first 96 Team Study participants were matched to the NHANES comparison cohort at least 1 : 2 (mean matches per TEAM participant was 2.13) after excluding 11 of the 107 who were taking diabetes medications to match NHANES eligibility criteria (including 4 with T1DM, 3 with T2DM, 3 with GDM, currently still on diabetes medication, and 1 with MODY). After matching, TEAM and NHANES participants were not appreciably different by age at screening, race, or sex. Groups did differ by several metabolic indicators (Table 2). While 32% of NHANES participants had normal BMI (<25 kg/m^2^), only 21% of TEAM participants had normal BMI (overall *P* = 0.04). Similarly, morbid obesity (≥40 kg/m^2^) was about 1.7 times as high among TEAM participants compared with NHANES participants (15% versus 9%). Both fasting glucose and two-hour glucose differed between NHANES and TEAM participants, though the results were somewhat less consistent. A normal two-hour glucose was present in 93% of NHANES participants and only 72% of TEAM participants and elevated among 2% versus 6% for NHANES and TEAM, respectively (*P* < 0.0001). For fasting glucose, three times the number of NHANES participants had impaired fasting glucose between 100 and 126 mg/dL (40% versus 14%), and a higher percentage of TEAM participants had normal fasting glucose < 126 mg/dL (82% versus 57%, *P* < 0.0001). In bivariate comparisons, diastolic blood pressure also differed significantly between NHANES and TEAM participants. Comparable findings were observed in multivariable analyses with and without adjustment for age (Figure 2).

Representations from the three groups of glucose profiles based on quartiles of fPC scores could be characterized as follows: group 1: both high mean and variability in glucose control across pregnancy; group 2: moderate mean levels with moderate variability; and group 3: low mean and variability across pregnancy. Mean levels of adult offspring BMI varied across profiles (35.6, 31.2, and 28.0 kg/m^2^, respectively, *P* value 0.05). However, their fasting and 2-hour plasma glucose as well as HbA1C did not vary by maternal glucose profiles (Table 3).

## 4. Discussion

We described the identification, recruitment, enrollment, and study completion of the first 107 participants of the TEAM Study. In addition, we observed that adult offspring born to mothers with type 1 diabetes during pregnancy were more likely to be obese and have impaired glucose metabolism as indicated by elevated two-hour glucose compared to an age-, sex-, and race-matched cohort. Finally, a profile of maternal glucose in pregnancy representing a high mean level with high variability of glucose across pregnancy was associated with obesity in a subset of participants. Overall, these results align with prior studies that have identified an association between exposure to glucose impairment *in utero* and adverse offspring metabolic outcomes. In addition, these results ideally frame the context for completing the TEAM Study with the aim of determining the timing in pregnancy that is most detrimental to development of metabolic impairment and how variability in the level of glucose exposure across pregnancy contributes to this impairment.

The importance of the fetal environment for adult health outcomes was popularized by the work of Barker who demonstrated that women and men whose own birth weights were low had an increased risk for coronary heart disease [28]. Following the “Barker hypothesis,” additional findings were found that not only low birth weight but also increased weights at birth were associated with adverse childhood and adult metabolic outcomes. For example, longitudinal studies in Pima Indians identified an association between small for gestational age, large for gestational age, and exposure to diabetes in pregnancy with type 2 diabetes later in life [29, 30]. Both obesity [31] and hyperglycemia in pregnancy [32, 33] have been associated with neonatal adiposity [34]. Research in this area was additionally guided by the Pedersen hypothesis, which suggested that fetal overgrowth was driven by placental transfer of maternal glucose, leading to the release of fetal insulin and, in turn, fetal macrosomia [35]. Evidence of fetal macrosomia and other short-term consequences of exposure to type 1 diabetes, such as still birth, major malformations, perinatal mortality, and preterm birth, have been demonstrated and broadly reproduced [21, 36–39].

Studies of the long-term offspring metabolic consequences of exposure to type 1 diabetes *in utero* are more sparse. However, results of existing studies are generally in line with our findings. For example, a study in Denmark of 160 offspring aged 18-27 years of women with type 1 diabetes identified a two-fold increased risk for overweight and 2.5-fold increased risk for metabolic syndrome compared with the background population [40]. Most striking is the background level of overweight (≥25 kg/m^2^) in each population, which was around 24% in Denmark and 65% in NHANES. Therefore, relative to background, differences between TEAM and NHANES participants were most evident at the highest levels of obesity with a nearly 2- and 3-fold increased risk for class II and class III obesity, respectively. In the same Danish cohort, comparisons of fasting glucose and two-hour glucose were also comparable to those of the TEAM Study cohort. As with the Danish study (5.2 versus 5.1 mmol/L; for offspring of women with type 1 diabetes versus control), we did not see appreciably higher levels of fasting glucose among offspring of type 1 diabetes; in fact, we observed lower levels among our offspring of type 1 diabetes compared to NHANES participants (5.0 versus 5.6 mmol/L; note: values are converted from mg/dL in Table 2 to mmol/L in order to compare with the Danish study). However, both studies observed larger differences compared with pregnancies without diabetes with mean two-hour glucose of 5.8 versus 5.3 mmol/L for the Danish study and 7.4 versus 5.6 mmol/L for the TEAM Study [6]. Despite several studies with comparable findings, we did identify one small study (*n* = 21) of young adult offspring aged 16 to 23 years born to women with type 1 diabetes which found no increase in blood glucose or anthropometric measures compared with no maternal history of diabetes [41]. The reasons for these findings are unclear but may be due to differences in exclusion criteria, for example, offspring with type 1 diabetes were excluded in the TEAM Study, due to comparison with the NHANES participants or due to variations in participation rates, potentially affecting their results.

The findings associating maternal glucose profiles in pregnancy with obesity in the offspring introduce the potential for identifying the critical windows and type of exposure (constant high exposure versus glucose excursions, for example) that are most detrimental to the developing fetus. Future analyses among the entire cohort will allow us to identify specific timing and variability associated with adverse metabolic and cardiac and nephrotic outcomes and refine these clinically relevant phenotypes.

A few limitations of the present analyses should be noted. First, it is unknown whether the NHANES participants were exposed to diabetes *in utero*. However, we can expect only a minority of the pregnancies complicated by diabetes, especially due to the age of the participants under study, and therefore, it would not have a strong effect on the results. In addition, any effect would likely underestimate the relative effect of *in utero* exposure for TEAM participants compared with NHANES participants. Also, for NHANES participants, we do not have detailed information on maternal blood glucose in pregnancy.

Overall, the results of the present analyses were in line with both our hypotheses and with the existing research. In addition, the results emphasize the need for future work that will elucidate the impact of timing and variability of maternal glycemia across pregnancy (the primary objectives of the TEAM Study). In addition to metabolic outcomes, the TEAM Study will identify risks for a wide range of cardiac, microvascular, cognitive, and nephrotic outcomes in these offspring, including subtle outcomes early in their natural history that may be amenable to secondary prevention. Diabetes in pregnancy affects more than 10% of pregnancies and is increasing in prevalence in the United States and therefore presents a considerable opportunity for prevention of these long-term consequences. With multiple daily measures of maternal glucose across pregnancy, the TEAM Study is uniquely positioned to answer these questions in the coming years.

## Figures and Tables

**Table 1:** The TEAM Study visit procedures, methods, lab tests, and assessments.

Assessment	Measurement
Pregnancy test	Urine pregnancy testing (for females)
Questionnaires	Health history, sociodemographic, physical activity, and sleep questionnaires
Cardiovascular	Endothelial & vascular function (Endo-Pat, FMD), blood pressure, brachial artery distensibility, augmentation index (AiX) and pulse wave velocity (PWV), carotid ultrasound, echocardiography
Renal	Creatinine, cystatin C, albumin, hepatic panel
Metabolic, diabetogenic	Oral glucose tolerance test (glucose, insulin), glucose-like peptide 1 (GLP-1), gastric inhibitory polypeptide (GIP), glucagon, C-peptide, hemoglobin A1c (HbA1C), lipids, islet cell antibodies (ICA), adiponectin, leptin, phospholipids, free fatty acids, high-sensitivity C-reactive protein (hsCRP), vitamin D, thyroid-stimulating hormone (TSH)
Nutrition	24-hour food recall (followed by 2 postvisit recalls) & block food frequency V3
Anthropometric	Hip, waist (iliac and midpoint) and sagittal abdominal diameter (SAD), dual X-ray absorptiometry (DXA)
Neurocognitive	Brief Symptom Index (BSI-18); Conners' Adult ADHD Ratings Scale (CAARS); Wechsler Abbreviated Scale of Intelligence, 2^nd^ edition (WASI-II); Social Responsiveness Scale (SRS-2); PedsQL

**Table 2:** Demographic and glycemic measures comparing TEAM participants to NHANES participants matched on age, race, and sex.

	NHANES participants (*N* = 213)	TEAM participants (*N* = 96)	*P* value^∗^
Age			
Mean (standard deviation)	31.8 (5.0)	32.0 (4.5)	0.78
Median (25^th^–75^th^ percentile)	32.0 (28.0-36.0)	32.2 (28.0-35.5)	
Race			
White	175 (82.2)	85 (88.5)	0.16
Black	38 (17.8)	11 (11.5)	
Male sex	118 (55.4)	52 (54.2)	0.84
BMI (kg/m^2^)			
Mean (standard deviation)	29.1 (7.5)	31.8 (8.1)	0.01
Median (25^th^–75^th^ percentile)	27.4 (23.8-33.0)	30.4 (26.4-35.1)	
Normal	68 (31.9)	20 (20.8)	0.04
Overweight	68 (31.9)	24 (25.0)	
Class I obesity	39 (18.3)	28 (29.2)	
Class II obesity	19 (8.9)	10 (10.3)	
Class III obesity	19 (8.9)	14 (14.6)	
Iliac waist circumference (cm)			
Mean (standard deviation)	98.3 (17.8)	102.2 (18.6)	0.09
Median (25^th^–75^th^ percentile)	95.9 (85.5-108.5)	98.7 (88.8-112.1)	
Systolic blood pressure (mmHg)			
Mean (standard deviation)	117.6 (12.4)	119.6 (11.5)	0.18
Median (25^th^–75^th^ percentile)	116.0 (109.3-124.7)	117.2 (111.8-126.2)	
Diastolic blood pressure (mmHg)			
Mean	69.4 (9.3)	75.2 (10.6)	<0.0001
Median	69.3 (62.7-76.0)	74.0 (68.0-83.0)	
Fasting glucose (mg/dL)			
Mean (standard deviation)	100.6 (23.9)	90.8 (23.0)	0.001
Median (25^th^–75^th^ percentile)	98.0 (92.0-103.0)	85.7 (80.2-96.2)	
<100	121 (56.8)	79 (82.3)	<0.0001
100-<126	86 (40.4)	13 (13.5)	
≥126	6 (2.8)	4 (4.2)	
Two-hour glucose (mg/dL)			
Mean (standard deviation)	100.8 (37.9)	133.5 (49.2)	<0.0001
Median (25^th^–75^th^ percentile)	95.0 (82.5-116.0)	124.3 (102.3-144.8)	
<140	171 (92.9)	69 (71.9)	<0.0001
140-<200	10 (5.4)	21 (21.9)	
≥200	3 (1.6)	6 (6.3)	
HbA1C (%)			
Mean (standard deviation)	5.3 (0.8)	5.4 (0.8)	0.24
Median (25^th^–75^th^ percentile)	5.2 (5.0-5.5)	5.3 (5.1-5.6)	
<5.7%	188 (88.7)	80 (83.3)	0.34
5.7-6.4%	20 (9.4)	12 (37.5)	
≥6.5%	4 (1.9)	4 (4.2)	

^∗^For continuous variables, difference between means and for categorical variables, chi-square.

**Table 3:** Metabolic outcomes by maternal glucose clusters representing glucose control across pregnancy for 56 TEAM Study participants.

Covariate		Maternal glucose clusters			
		1 (*N* = 9)	2 (*N* = 32)	3 (*N* = 15)	Parametric *P* value^∗^
BMI categories	Normal	1 (11.11)	6 (18.75)	2 (13.33)	0.25
	Overweight	0 (0)	6 (18.75)	6 (40)	
	Obesity class I	2 (22.22)	12 (37.5)	4 (26.67)	
	Obesity class II	2 (22.22)	3 (9.38)	1 (6.67)	
	Obesity class III	4 (44.44)	5 (15.63)	2 (13.33)	
BMI (kg/m^2^)		9	32	15	0.05
	Mean	40.0	32.5	31.5	
	Median	35.6	31.2	28.0	
HbA1C		9	32	15	0.26
	Mean	5.4	5.7	5.3	
	Median	5.5	5.5	5.3	
Fasting plasma glucose (mg/dL)		9	32	15	0.68
	Mean	90.7	95.2	88.0	
	Median	86.1	89.1	86.0	
2-hour plasma glucose (mg/dL)		9	32	15	0.49
	Mean	125.3	141.9	123.7	
	Median	124.5	132.4	114	

^∗^The parametric *P* value is calculated by ANOVA for numerical covariates and the chi-square test for categorical covariates.

## Data Availability

The TEAM Study data used to support the findings of this study may be released upon the application of the TEAM Study data and specimen request form. Contact Dr. Jane Khoury for details at jane.khoury@cchmc.org.

## References

[1] Dwyer-Lindgren L., Mackenbach J. P., van Lenthe F. J., Flaxman A. D., Mokdad A. H. (2016). Diagnosed and Undiagnosed Diabetes Prevalence by County in the U.S., 1999–2012. *Diabetes Care*.

[2] Control CfD, Prevention (2011). *National diabetes fact sheet: national estimates and general information on diabetes and prediabetes in the United States, 2011*.

[3] Lawrence J. M., Contreras R., Chen W., Sacks D. A. (2008). Trends in the prevalence of preexisting diabetes and gestational diabetes mellitus among a racially/ethnically diverse population of pregnant women, 1999-2005. *Diabetes Care*.

[4] Pettitt D. J., Nelson R. G., Saad M. F., Bennett P. H., Knowler W. C. (1993). Diabetes and obesity in the offspring of Pima Indian women with diabetes during pregnancy. *Diabetes Care*.

[5] Rodrigues S., Ferris A. M., Perez-Escamilla R., Backstrand J. R. (1998). Obesity among offspring of women with type 1 diabetes. *Clinical and Investigative Medicine*.

[6] Clausen T. D., Mathiesen E. R., Hansen T. (2008). High prevalence of type 2 diabetes and pre-diabetes in adult offspring of women with gestational diabetes mellitus or type 1 diabetes: the role of intrauterine hyperglycemia. *Diabetes Care*.

[7] Plagemann A., Harder T., Kohlhoff R., Rohde W., Dorner G. (1997). Overweight and obesity in infants of mothers with long-term insulin- dependent diabetes or gestational diabetes. *International Journal of Obesity and Related Metabolic Disorders*.

[8] Sobngwi E., Boudou P., Mauvais-Jarvis F. (2003). Effect of a diabetic environment in utero on predisposition to type 2 diabetes. *Lancet*.

[9] Weiss P. A., Scholz H. S., Haas J., Tamussino K. F., Seissler J., Borkenstein M. H. (2000). Long-term follow-up of infants of mothers with type 1 diabetes: evidence for hereditary and nonhereditary transmission of diabetes and precursors. *Diabetes Care*.

[10] Plagemann A., Harder T., Kohlhoff R., Rohde W., Dorner G. (1997). Glucose tolerance and insulin secretion in children of mothers with pregestational IDDM or gestational diabetes. *Diabetologia*.

[11] Franks P. W., Looker H. C., Kobes S. (2006). Gestational glucose tolerance and risk of type 2 diabetes in young Pima Indian offspring. *Diabetes*.

[12] Ievins R., Roberts S. E., Goldacre M. J. (2007). Perinatal factors associated with subsequent diabetes mellitus in the child: record linkage study. *Diabetic Medicine*.

[13] Silverman B. L., Rizzo T. A., Cho N. H., Metzger B. E. (1998). Long-term effects of the intrauterine environment. The Northwestern University Diabetes in Pregnancy Center. *Diabetes Care*.

[14] Lee H., Jang H. C., Park H. K., Cho N. H. (2007). Early manifestation of cardiovascular disease risk factors in offspring of mothers with previous history of gestational diabetes mellitus. *Diabetes Research and Clinical Practice*.

[15] Fraser A., Lawlor D. A. (2014). Long-term health outcomes in offspring born to women with diabetes in pregnancy. *Current Diabetes Reports*.

[16] Bytoft B., Knorr S., Vlachova Z. (2016). Long-term cognitive implications of intrauterine hyperglycemia in adolescent offspring of women with type 1 diabetes (the EPICOM study). *Diabetes Care*.

[17] Kitzmiller J. L., Buchanan T. A., Siri K., Combs A. C., Ratner R. E. (1996). Pre-conception care of diabetes, congenital malformations, and spontaneous abortions. *Diabetes Care*.

[18] Gabbay-Benziv R., Reece E. A., Wang F., Yang P. (2015). Birth defects in pregestational diabetes: defect range, glycemic threshold and pathogenesis. *World Journal of Diabetes*.

[19] Ballard J. L., Holroyde J., Tsang R. C., Chan G., Sutherland J. M., Knowles H. C. (1984). High malformation rates and decreased mortality in infants of diabetic mothers managed after the first trimester of pregnancy (1956-1978). *American Journal of Obstetrics and Gynecology*.

[20] Tennant P. W., Glinianaia S. V., Bilous R. W., Rankin J., Bell R. (2014). Pre-existing diabetes, maternal glycated haemoglobin, and the risks of fetal and infant death: a population-based study. *Diabetologia*.

[21] Macintosh M. C., Fleming K. M., Bailey J. A. (2006). Perinatal mortality and congenital anomalies in babies of women with type 1 or type 2 diabetes in England, Wales, and Northern Ireland: population based study. *BMJ*.

[22] McElvy S. S., Miodovnik M., Rosenn B. (2000). A focused preconceptional and early pregnancy program in women with type 1 diabetes reduces perinatal mortality and malformation rates to general population levels. *The Journal of Maternal-Fetal Medicine*.

[23] Drehmer M., Duncan B. B., Kac G., Schmidt M. I. (2013). Association of second and third trimester weight gain in pregnancy with maternal and fetal outcomes. *PLoS One*.

[24] Boston R. C. S. D., Stefanovski D., Moate P. J., Sumner A. E., Watanabe R. M., Bergman R. N. (2003). MINMOD millennium: a computer program to calculate glucose effectiveness and insulin sensitivity from the frequently sampled intravenous glucose tolerance test. *Diabetes Technology and Therapeutics*.

[25] Brady L., Gower B., Lovegrove S., Williams C., Lovegrove J. (2004). Revised QUICKI provides a strong surrogate estimate of insulin sensitivity when compared with the minimal model. *International Journal of Obesity*.

[26] Bergstralh E., Kosanke J. (1995). *Computerized matching of controls: section of biostatistics technical report 56*.

[27] Szczesniak R. D., Li D., Duan L. L., Altaye M., Miodovnik M., Khoury J. C. (2016). Longitudinal patterns of glycemic control and blood pressure in pregnant women with type 1 diabetes mellitus: phenotypes from functional data analysis. *American Journal of Perinatology*.

[28] Barker D. J. (1995). Fetal origins of coronary heart disease. *BMJ*.

[29] Dabelea D., Hanson R., Bennett P. H., Roumain J., Knowler W. C., Pettitt D. (1998). Increasing prevalence of type II diabetes in American Indian children. *Diabetologia*.

[30] Pettitt D. J., Baird H. R., Aleck K. A., Bennett P. H., Knowler W. C. (1983). Excessive obesity in offspring of Pima Indian women with diabetes during pregnancy. *The New England Journal of Medicine*.

[31] Catalano P. M. (2003). Obesity and pregnancy--the propagation of a viscous cycle?. *The Journal of Clinical Endocrinology and Metabolism*.

[32] Catalano P. M., Kirwan J. P., Haugel-de Mouzon S., King J. (2003). Gestational diabetes and insulin resistance: role in short- and long-term implications for mother and fetus. *The Journal of Nutrition*.

[33] Lowe L. P., Metzger B. E., Dyer A. R. (2012). Hyperglycemia and adverse pregnancy outcome (HAPO) study: associations of maternal A1C and glucose with pregnancy outcomes. *Diabetes*.

[34] Plagemann A. (2005). Perinatal programming and functional teratogenesis: impact on body weight regulation and obesity. *Physiology & Behavior*.

[35] Pedersen J. (1954). Weight and length at birth of infants of diabetic mothers. *European Journal of Endocrinology*.

[36] Persson M., Norman M., Hanson U. (2009). Obstetric and perinatal outcomes in type 1 diabetic pregnancies: a large, population-based study. *Diabetes Care*.

[37] Jensen D. M., Damm P., Moelsted-Pedersen L. (2004). Outcomes in type 1 diabetic pregnancies: a nationwide, population-based study. *Diabetes Care*.

[38] Lepercq J. (2003). French multicentric survey of outcome of pregnancy in women with pregestational diabetes. *Diabetes Care*.

[39] Kim S. Y., Kotelchuck M., Wilson H. G., Diop H., Shapiro-Mendoza C. K., England L. J. (2015). Prevalence of Adverse Pregnancy Outcomes, by Maternal Diabetes Status at First and Second Deliveries, Massachusetts, 1998–2007. *Preventing Chronic Disease*.

[40] Clausen T. D., Mathiesen E. R., Hansen T. (2009). Overweight and the metabolic syndrome in adult offspring of women with diet-treated gestational diabetes mellitus or type 1 diabetes. *The Journal of Clinical Endocrinology & Metabolism*.

[41] Cross J., Brennan C., Gray T. (2009). Absence of telomere shortening and oxidative DNA damage in the young adult offspring of women with pre-gestational type 1 diabetes. *Diabetologia*.

